# Septicæmia Resulting from a Leech Bite

**Published:** 1881-11

**Authors:** 


### SEPTICÆMIA RESULTING FROM A LEECH-BITE.

The *Gazz. Med. Ital.* quotes the following case: A Bernese had been suffering from toothache for several days, and to relieve it he followed out the advice of a dentist by applying a leech to the gum. Two hours later the pain increased, and the lip became the seat of an inflammation which extended to the cheek, neck and breast. The following day the head was swollen, respiration was difficult, and the patient was in high fever. A few hours later he was attacked with delirium tremor and convulsive vomiting. He died during the night of the second day from septicæmia, as demonstrated by the autopsy. The leech-bite was larger than the ordinary, and its borders were black and gangrenous. A poison of unknown nature had evidently gained access to the system through this wound.—*N. Y. Med. Record.*

